# A LODDS and PNI–based personalized prognostic model for patients with gastric cancer receiving neoadjuvant chemotherapy

**DOI:** 10.3389/fnut.2026.1861719

**Published:** 2026-08-17

**Authors:** Zhengzhao Wang, Wenzhi Wu, Zongsheng Sun, Mingyu Yang, Jingnong Liu, Yuandi Wang, Xinyu Long, Yunfei Zhang, Maoshen Zhang, Xianxiang Zhang, Wenchang Yang, Dongsheng Wang

**Affiliations:** Department of Gastrointestinal Surgery, Affiliated Hospital of Qingdao University, Qingdao, China

**Keywords:** gastric cancer, LODDS, neoadjuvant chemotherapy, nutrition, prognosis

## Abstract

**Background:**

This study aimed to develop and validate a risk stratification model incorporating the log odds of positive lymph nodes (LODDS) and the prognostic nutritional index (PNI) to predict outcomes in patients with locally advanced gastric cancer (LAGC) undergoing neoadjuvant chemotherapy (NAC).

**Methods:**

A retrospective cohort of patients with NAC–LAGC who underwent radical gastrectomy between January 2016 and January 2025 was enrolled. The cohort was randomly split into a training set and a validation set at a 7:3 ratio. Independent risk factors were identified through univariable and multivariable Cox regression analyses in the training set. An individualized nomogram for progression–free survival (PFS) and a risk stratification model were subsequently developed. Model performance was evaluated using the area under the receiver operating characteristic (ROC) curve (AUC), calibration curves, and decision curve analysis (DCA), and was further validated in the validation set.

**Results:**

A total of 450 patients were included. In the training set, 255 (79.2%) were male, and 67 (20.8%) were female; 96 (29.8%) were under 60 years, and 226 (70.2%) were 60 years or older. Multivariable analysis identified LODDS, PNI, age, tumor regression grade (TRG), and ypT stage as independent risk factors for PFS. The nomogram based on LODDS outperformed the ypN stage–based nomogram (AUCs at 3, 4, and 5 years: 0.916 vs. 0.888, 0.917 vs. 0.867, and 0.935 vs. 0.876, respectively; all *P* < 0.05 by Delong test). To enhance risk stratification, a prognostic model (LTTAP) was developed, demonstrating robust predictive performance with all AUCs exceeding 0.850 and a concordance index (C–index) of 0.826. DCA further confirmed that LTTAP provided a meaningful clinical net benefit for NAC–LAGC patients.

**Conclusion:**

The LTTAP model integrating LODDS and PNI offers strong prognostic value for patients with NAC–LAGC.

## 1 Introduction

Gastric cancer is one of the leading causes of cancer–related mortality worldwide (1). Neoadjuvant chemotherapy (NAC) combined with radical gastric cancer resection has become the standard treatment regimen for patients with locally advanced gastric cancer (LAGC) (2). While this approach significantly facilitates tumor downstaging and eradicates micro–metastases, there remains considerable heterogeneity in patient prognosis, influenced by both the tumor's biological characteristics and individual patient differences, even with standardized treatment (3, 4).

Host–related factors, particularly alterations in inflammatory and nutritional status following NAC, play a critical role in recurrence. Chemotherapy–induced gastrointestinal toxicity can directly impair nutritional intake, while treatment–induced catabolic responses and systemic inflammation may lead to hypoalbuminemia, muscle wasting, and immune dysfunction (5, 6). These changes not only reflect a decline in physiological reserves but also contribute to postoperative recurrence by weakening antitumor immunity, reducing treatment tolerance, and fostering an adverse tumor microenvironment. Therefore, a comprehensive preoperative nutritional assessment is essential for accurate prognostic risk stratification and guiding perioperative nutritional interventions. Previous studies have demonstrated that inflammatory–nutritional markers such as PNI and NLR provide significant clinical value in predicting prognosis and recurrence across a variety of malignant tumors (7, 8).

Pathological TNM staging (ypTNM) following neoadjuvant treatment is strongly correlated with disease–free survival (DFS) and overall survival (OS) in patients, serving as the foundation for postoperative prognosis assessment and risk stratification in gastric cancer. It is extensively used for postoperative risk evaluation and follow–up management (9, 10). Tumor regression grading (TRG) is recognized as a key indicator for assessing the response to NAC, reflecting the tumor's biological sensitivity (11). However, in the context of NAC, the predictive power of traditional staging systems is often limited. Variations in the scope of lymph node dissection and differences in pathological sampling can lead to “stage migration” in N staging (12, 13), while anatomical staging alone fails to capture the tumor's biological response to treatment or the host's systemic status. To address the limitations of ypN staging, recent studies have proposed the use of log odds of positive lymph nodes (LODDS) in prognosis models for rectal cancer patients (14, 15). Recently, LODDS has also been increasingly applied in the prognosis evaluation of gastric cancer patients, with several studies demonstrating its effectiveness in predicting gastric cancer outcomes (16).

This study integrated LODDS and inflammatory–nutritional indicators in patients with LAGC who underwent NAC followed by radical resection. Additionally, an individualized risk–stratification model for recurrence was developed and validated. Our findings provide a decision–support tool for precise identification of high–risk postoperative patients, optimizing follow–up strategies, and facilitating personalized management.

## 2 Methods

### 2.1 Patients and research design

**Figure 1:**
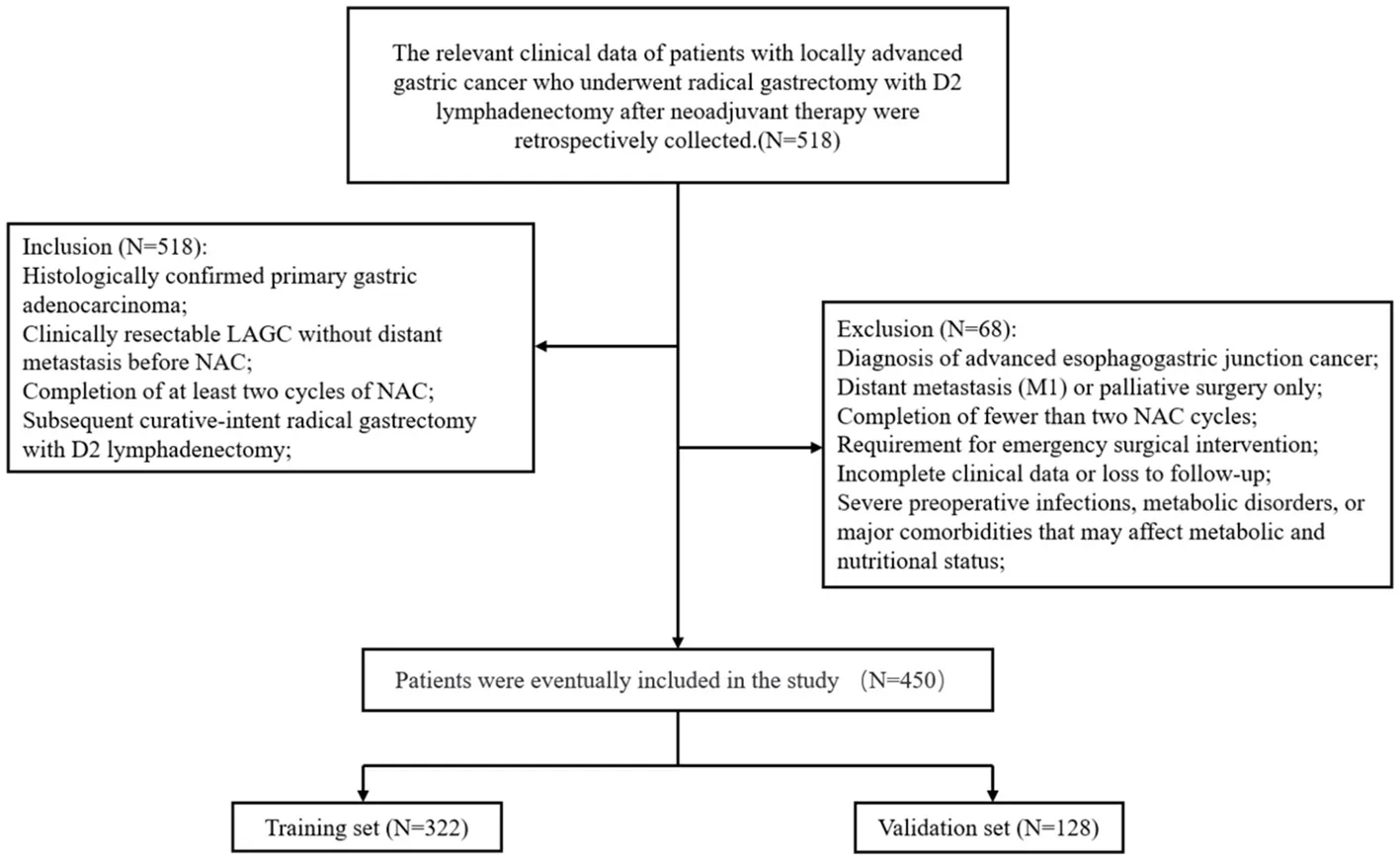
Flowchart of patient inclusion and exclusion in the present study.

This study retrospectively collected NAC-LAGC patients at the Affiliated Hospital of Qingdao University between January 2016 and January 2025. The inclusion criteria were as follows: (1) histologically confirmed primary gastric adenocarcinoma; (2) clinically resectable LAGC without distant metastasis before NAC; (3) completion of at least two cycles of NAC; and (4) subsequent curative-intent radical gastrectomy with D2 lymphadenectomy. Patients who experienced treatment-related adverse events during or after completing at least two cycles of NAC remained eligible for inclusion, provided that they subsequently underwent curative-intent radical gastrectomy. The exclusion criteria were as follows: (1) diagnosis of advanced esophagogastric junction cancer; (2) distant metastasis (M1) or palliative surgery only; (3) completion of fewer than two NAC cycles; (4) requirement for emergency surgical intervention; (5) incomplete clinical data or loss to follow–up, and (6) severe preoperative infections, metabolic disorders, or major comorbidities that may affect metabolic and nutritional status (e.g., chronic kidney disease, liver cirrhosis, obesity, or poorly controlled diabetes mellitus). The main NAC regimens were SOX (S−1 plus oxaliplatin) and FLOT (fluorouracil, leucovorin, oxaliplatin, and docetaxel). According to the 5th edition of the Japanese Gastric Cancer Treatment Guidelines, patients underwent radical gastrectomy and D2 lymphadenectomy approximately 4–6 weeks after completing all NAC cycles (17). All procedures were performed by experienced gastrointestinal surgeons according to standardized protocols. A total of 450 eligible patients were ultimately enrolled after applying the inclusion and exclusion criteria. The final cohort was randomly divided into a training set and a validation set at a ratio of 7:3. The study complied with the Declaration of Helsinki and was approved by the Ethics Committee of the Affiliated Hospital of Qingdao University (approval no. QYFY WZLL 50021). A schematic overview of the study design is presented in Figure 1.

### 2.2 Data collection and follow-up

This study collected comprehensive baseline clinical data, including gender, age, ypTNM staging, total number of lymph nodes detected (TLN), number of metastatic positive lymph nodes (PLN), BMI before and after NAC, tumor size, and others. Postoperative specimens were evaluated by professional pathologists, recording tumor size, pathological staging, and TRG. Laboratory tests performed within one week prior to surgery were included, such as CA19–9, CA12–5, CEA, neutrophil count, lymphocyte count, hemoglobin, albumin, platelet count, and globulin. The calculation methods for inflammation–nutrition–related indices were as follows: NLR = N/L, PLR = P/L, PNI = albumin (g/L) + 5 × L (10^9^/L), NRI = 1.519 × albumin (g/L) + 41.7 × (post–NAC weight / pre–NAC weight). The specific calculation formula for LODDS was as follows: LODDS = ln[(pLN+0.5)/(TLN–pLN+0.5)] (18). Follow–up was conducted through outpatient visits or telephone interviews, following a standardized schedule: every 3 months for the first two years post–operation, every 6 months between years 2 and 5, and then annually, with follow–up ending before October 2025. Progression–free survival (PFS) was defined as the interval from surgery to the earliest disease progression (19).

### 2.3 Data preprocessing and feature selection

All independent variables were preprocessed before analysis. Continuous variables were Z–score standardized by calculating the mean and standard deviation on the training set, with the same standardization parameters applied to the validation set. Categorical variables were converted to dummy variables using one–hot encoding. Age and albumin levels were dichotomized based on clinically commonly used thresholds (60 years and 35 g/L) (20, 21). The cutoff values for LODDS were determined through exploratory analysis on the training set data using the “X–tile” package. According the optimal grouping thresholds, being −3.65 and −0.755, all samples were divided into three groups. On the training set, univariate Cox regression analysis was conducted firstly, and the variables with a *p*–value less than 0.10 were selected for further multivariate analysis.

### 2.4 Construction and validation of prognostic models for PFS

Based on the variables identified through univariate Cox analysis, two multivariate Cox regression models were developed. Nomogram models for progression–free survival (PFS) were then constructed for both models. Internal validation was performed using 1,000 bootstrap resampling to assess the risk of overfitting and evaluate the robustness of the models. Harrell's concordance index (C-index) was calculated to evaluate the overall discriminative ability of each prognostic model, with a higher C-index indicating better discrimination between patients with different risks of disease progression. Calibration curves were plotted in the training set to assess the potential clinical application value of the predictive models. The model's discriminative ability was evaluated through receiver operating characteristic (ROC) curve analysis, with the area under the curve (AUC) serving as an indicator of prediction accuracy. Predictive performance was subsequently re–evaluated in the validation set, with decision curve analysis (DCA) performed to further assess clinical applicability.

### 2.5 Development and evaluation of the prognostic risk stratification model

The risk score for each patient was calculated based on the regression coefficients of each variable in the original nomogram model. Optimal cutoff values were determined using the “X–tile” package in R software, and patients were categorized into four risk levels: I, II, III, and IV. Kaplan–Meier survival curves were used to compare prognostic differences across different risk groups. To further illustrate the expression distribution of model variables in each risk group, corresponding heatmaps were generated. A heatmap was also constructed based on recurrence status and recurrence time data, enabling a visual comparison of clinical outcomes across the risk groups. Finally, ROC curves and DCA curves were plotted to evaluate the predictive performance and clinical applicability of the prognostic risk stratification model.

### 2.6 Statistical analysis

All statistical analyses were conducted using R software (version 4.4.2). Continuous variables are reported as mean ± standard deviation (SD) or median with interquartile range (IQR), according to their distribution, and were compared using the independent-samples *t*-test or the corresponding nonparametric test, as appropriate. Categorical variables are presented as frequencies and percentages [n (%)] and were compared using the chi-square test. Data preprocessing was performed with the “dplyr” package. The “survival” package was used for Cox proportional hazards regression and Kaplan–Meier survival analysis. Optimal cutoff values for risk stratification were identified using the “X-tile” package. Nomogram construction and calibration curve generation were performed using the “rms” package. Model discrimination was evaluated by ROC curve analysis, with AUC values calculated accordingly. DCA was performed using the “rmda” package. A two-sided *P*-value < 0.05 was considered statistically significant.

## 3 Results

### 3.1 Baseline characteristics

A total of 450 patients were included in this study, with baseline characteristics summarized in Table 1. The cohort was divided into a training set (*n* = 322) and an internal validation set (*n* = 128). In the training set, 255 patients (79.2%) were male, while in the validation set, 95 patients (74.2%) were male. In the training set, 96 patients (29.8%) were under 60 years of age, compared to 42 patients (32.8%) in the validation set. Among the 450 patients, 304 (67.7%) had positive lymph nodes, while 146 (32.3%) did not. In the training set, 54 patients (16.8%) had a TRG of 0, indicating complete remission, whereas 32 patients (25.0%) in the validation set achieved a TRG of 0. The distribution of ypTNM staging in the training set was as follows: 80 patients (24.8%) in stage I, 72 patients (22.4%) in stage II, and 170 patients (52.8%) in stage III. In the validation set, 35 patients (27.3%) were in stage I, 35 patients (27.3%) were in stage II, and 58 patients (45.3%) were in stage III. In the training set, 162 patients (50.3%) received postoperative adjuvant therapy, compared with 66 patients (51.6%) in the validation set. The corresponding numbers of patients who did not receive postoperative adjuvant therapy were 160 (49.7%) and 62 (48.4%), respectively. No significant differences in baseline characteristics were found between the two sets (*p* > 0.05).

**Table 1:** Baseline characteristics of the total, training, and validation cohorts.

Variable	Total cohort (*n* = 450)	Training cohort (*n* = 322)	Validation cohort (*n* = 128)	*P*-value
Sex, *n* (%)				0.252
Female	100 (22.2)	67 (20.8)	33 (25.8)	
Male	350 (77.8)	255 (79.2)	95 (74.2)	
Age, *n* (%)				0.534
< 60	138 (30.7)	96 (29.8)	42 (32.8)	
≥60	312 (69.3)	226 (70.2)	86 (67.2)	
PreBMI, Mean (SD)	26.56 (2.52)	26.54 (2.45)	26.60 (2.71)	0.820
PostBMI, Mean (SD)	25.60 (2.33)	25.60 (2.25)	25.60 (2.52)	0.979
Tumor size, Mean (SD)	4.22 (2.24)	4.19 (2.24)	4.29 (2.24)	0.660
CEA, Mean (SD)	27.10 (131.71)	28.62 (150.01)	23.28 (66.54)	0.602
CA199, Mean (SD)	167.74 (740.53)	194.84 (808.95)	99.56 (527.41)	0.143
CA125, Mean (SD)	22.47 (32.73)	21.67 (28.83)	24.47 (41.00)	0.482
Thrombocyte, mean (SD)	230.30 (58.61)	231.14 (57.47)	228.20 (61.57)	0.642
Glob, mean (SD)	25.65 (3.83)	25.64 (3.66)	25.68 (4.26)	0.930
Neutrophil, mean (SD)	4.06 (1.86)	4.05 (1.97)	4.07 (1.56)	0.904
Lymphocyte, mean (SD)	1.75 (0.62)	1.73 (0.63)	1.78 (0.58)	0.387
Monocyte, mean (SD)	0.47 (0.18)	0.46 (0.19)	0.49 (0.18)	0.076
Postoperative adjuvant treatment, *n* (%)				0.811
Yes	218 (50.7)	162 (50.3)	66 (51.6)	
No	232 (49.3)	160 (49.7)	62 (48.4)	
NLR, *n* (%)				0.551
< 2.05	187 (41.6)	131 (40.7)	56 (43.8)	
≥2.05	263 (58.4)	191 (59.3)	72 (56.2)	
Alb, *n* (%)				0.445
>35	35 (7.8)	27 (8.4)	8 (6.2)	
≤ 35	415 (92.2)	295 (91.6)	120 (93.8)	
PLR, *n* (%)				0.482
< 170.9	356 (79.1)	252 (78.3)	104 (81.2)	
≥170.9	94 (20.9)	70 (21.7)	24 (18.8)	
PNI, *n* (%)				0.281
>48.9	193 (42.9)	133 (41.3)	60 (46.9)	
≤ 48.9	257 (57.1)	189 (58.7)	68 (53.1)	
NRI, *n* (%)				0.852
< 103.1	250 (55.6)	178 (55.3)	72 (56.2)	
≥103.1	200 (44.4)	144 (44.7)	56 (43.8)	
PLN, *n* (%)				0.572
< 1	146 (32.4)	107 (33.2)	39 (30.5)	
≥1	304 (67.6)	215 (66.8)	89 (69.5)	
ypT stage, *n* (%)				0.152
T1–T2	170 (37.8)	115 (35.7)	55 (43.0)	
T3–T4	280 (62.2)	207 (64.3)	73 (57.0)	
LODDS, *n* (%)				0.544
≤ -3.65	217 (48.2)	150 (46.6)	67 (52.3)	
−3.65 to −0.755	141 (31.3)	104 (32.3)	37 (28.9	
≥-0.755	92 (20.4)	68 (21.1)	24 (18.8)	
TRG, *n* (%)				0.221
0	86 (19.1)	54 (16.8)	32 (25.0)	
1	108 (24.0)	78 (24.2)	30 (23.4)	
2	168 (37.3)	123 (38.2)	45 (35.2)	
3	88 (19.6)	67 (20.8)	21 (16.4)	
ypN stage, *n* (%)				0.078
0	118 (26.2)	81 (25.2)	37 (28.9)	
1	102 (22.7)	65 (20.2)	37 (28.9)	
2	122 (27.1)	91 (28.3)	31 (24.2)	
3	108 (24.0)	85 (26.4)	23 (18.0)	
ypTNM, *n* (%)				0.334
I	115 (25.6)	80 (24.8)	35 (27.3)	
II	107 (23.8)	72 (22.4)	35 (27.3)	
III	228 (50.7)	170 (52.8)	58 (45.3)	

^*^P < 0.05. SD, Standard Deviation; BMI, body mass index; CEA, carcinoembryonic antigen; CA199, Carbohydrate Antigen 19-9; CA125, cancer antigen 125; Glob, globulin; NRS, nutritional risk score; Alb, albumin; NLR, neutrophil-lymphocyte ratio; PLR, platelet-lymphocyte ratio; PNI, prognostic nutritional index; NRI, nutritional risk index; PLN, Positive Lymph Nodes; ypT stage, post-therapeutic pathological tumor stage; ypN stage, post-therapeutic pathological node stage; TRG, tumor regression grade; TRG, tumor regression grade; LODDS, log odds of positive lymph nodes; ypTNM stage, post-therapeutic pathological TNM stage.

### 3.2 Feature selection

**Figure 2:**
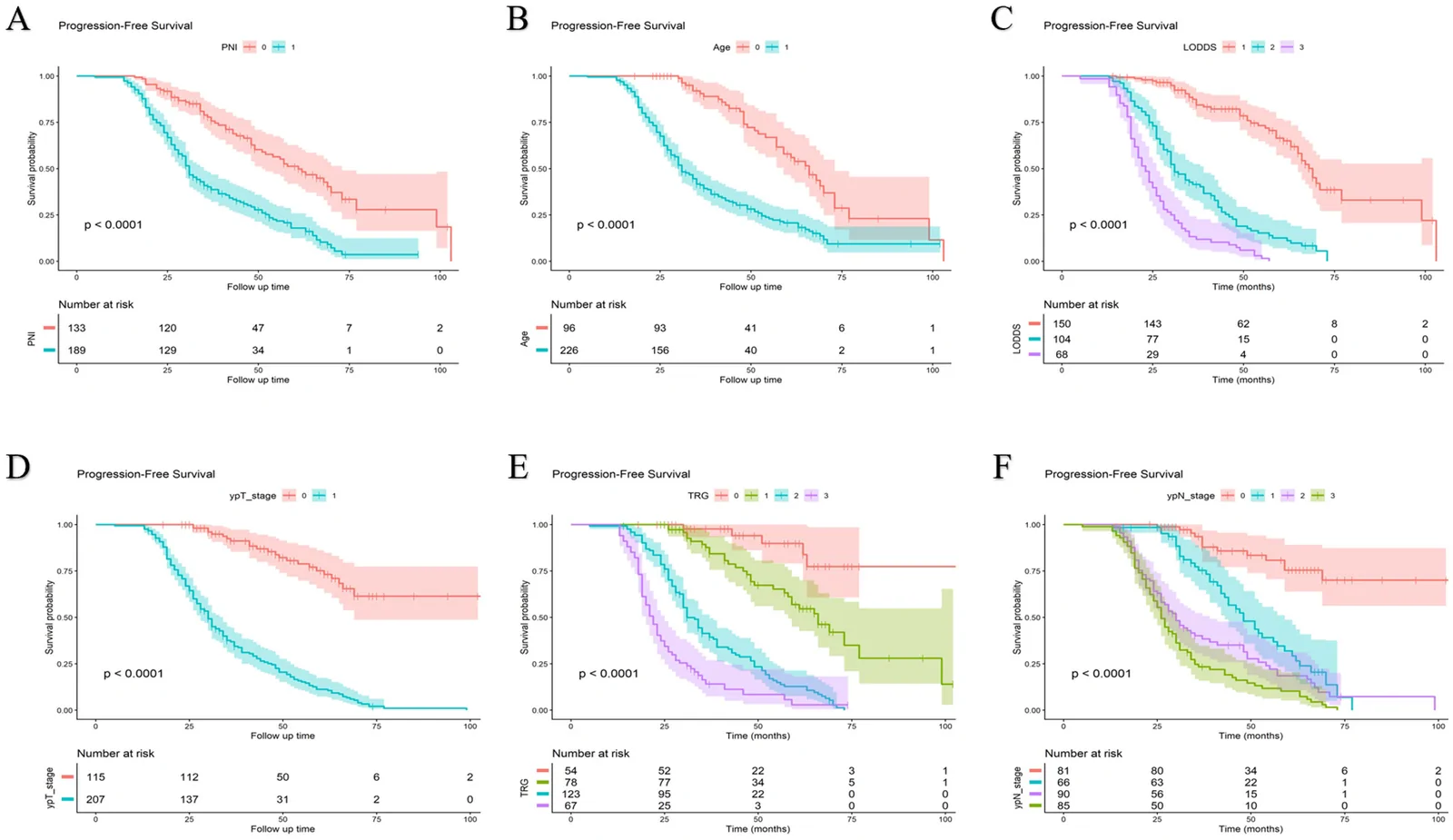
Kaplan-Meier survival analysis was performed based on six characteristics. PNI, Prognostic Nutritional Index; LODDS, Log odds of positive lymph nodes; ypT_stage, post-therapeutic pathological tumor stage; TRG, Tumor Regression Grade; ypN_stage, post-therapeutic pathological node stage.

In the training set, univariate Cox regression analysis was first performed on all categorical and continuous variables, resulting in the retention of six variables with *p* < 0.10 for multivariate Cox regression analysis. The univariate analysis identified age, LODDS, ypT stage, ypN stage, TRG, and PNI as significantly associated with PFS (*p* < 0.10) (Table 2). Kaplan–Meier survival analysis further revealed that advanced age, low PNI, higher TRG scores, high ypT stage, high ypN stage, and high LODDS were all significantly associated with an increased risk of recurrence (*p* < 0.001) (Figures 2A–F).

**Table 2:** Univariable Cox proportional hazards regression analysis of progression-free survival (PFS) in the training cohort of patients with NAC-LAGC.

Variable	Univariate analysis		
	HR	95% CI	*P*-value
Age			
< 60	–	–	–
≥60	3.381	2.366–4.830	<0.001^*^
LODDS			
≤ -3.65	–	–	–
−3.65 to −0.755	5.194	3.600–7.494	<0.001^*^
≥−0.755	12.471	8.322–18.689	<0.001^*^
ypT stage			
T1–T2	–	–	–
T3–T4	8.496	5.483–13.164	<0.001^*^
ypN stage			
N0	–	–	–
N1	5.033	2.694–9.402	<0.001^*^
N2	8.779	4.840–15.923	<0.001^*^
N3	13.854	7.662–25.050	<0.001^*^
TRG			
0	–	–	–
1	4.049	1.577–10.395	0.004^*^
2	16.678	6.775–41.060	<0.001^*^
3	36.299	14.463–91.103	<0.001^*^
PNI			
>48.9	–	–	–
≤ 48.9	2.966	2.174–4.047	<0.001^*^
NLR			
< 2.05	–	–	–
≥2.05	0.829	0.626–1.098	0.190
Alb			
>35	–	–	–
≤ 35	0.792	0.507–1.237	0.305
NRI			
< 103.1	–	–	–
≥103.1	1.131	0.857–1.493	0.383
PLR			
< 170.9	–	–	–
≥170.9	1.110	0.805–1.532	0.524

^*^P <0.05 was considered statistically significant.

### 3.3 Evaluation of the PFS prognostic model and survival analysis

**Figure 3:**
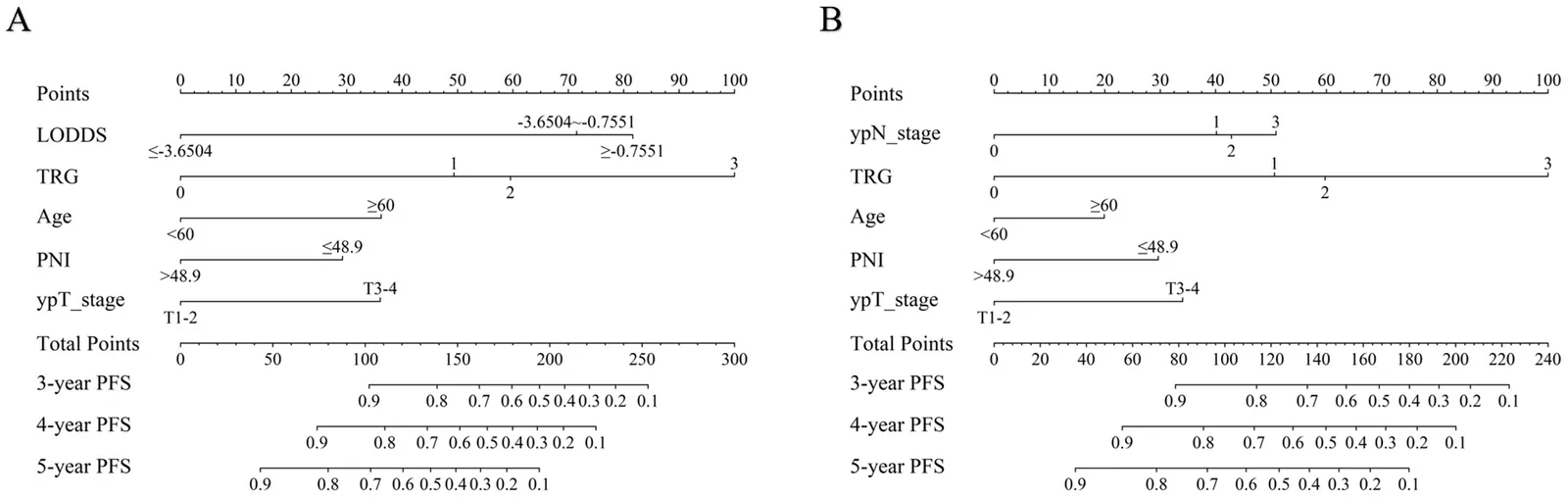
Nomograms for predicting postoperative recurrence risk in patients with NAC-LAGC. **(A)** Nomogram of Model 1 based on LODDS. **(B)** Nomogram of Model 2 based on ypN stage. PNI, Prognostic Nutritional Index; LODDS, Log odds of positive lymph nodes; ypT_stage, post-therapeutic pathological tumor stage; TRG, Tumor Regression Grade; ypN_stage, post-therapeutic pathological node stage; PFS, Progression-Free Survival.

**Figure 4:**
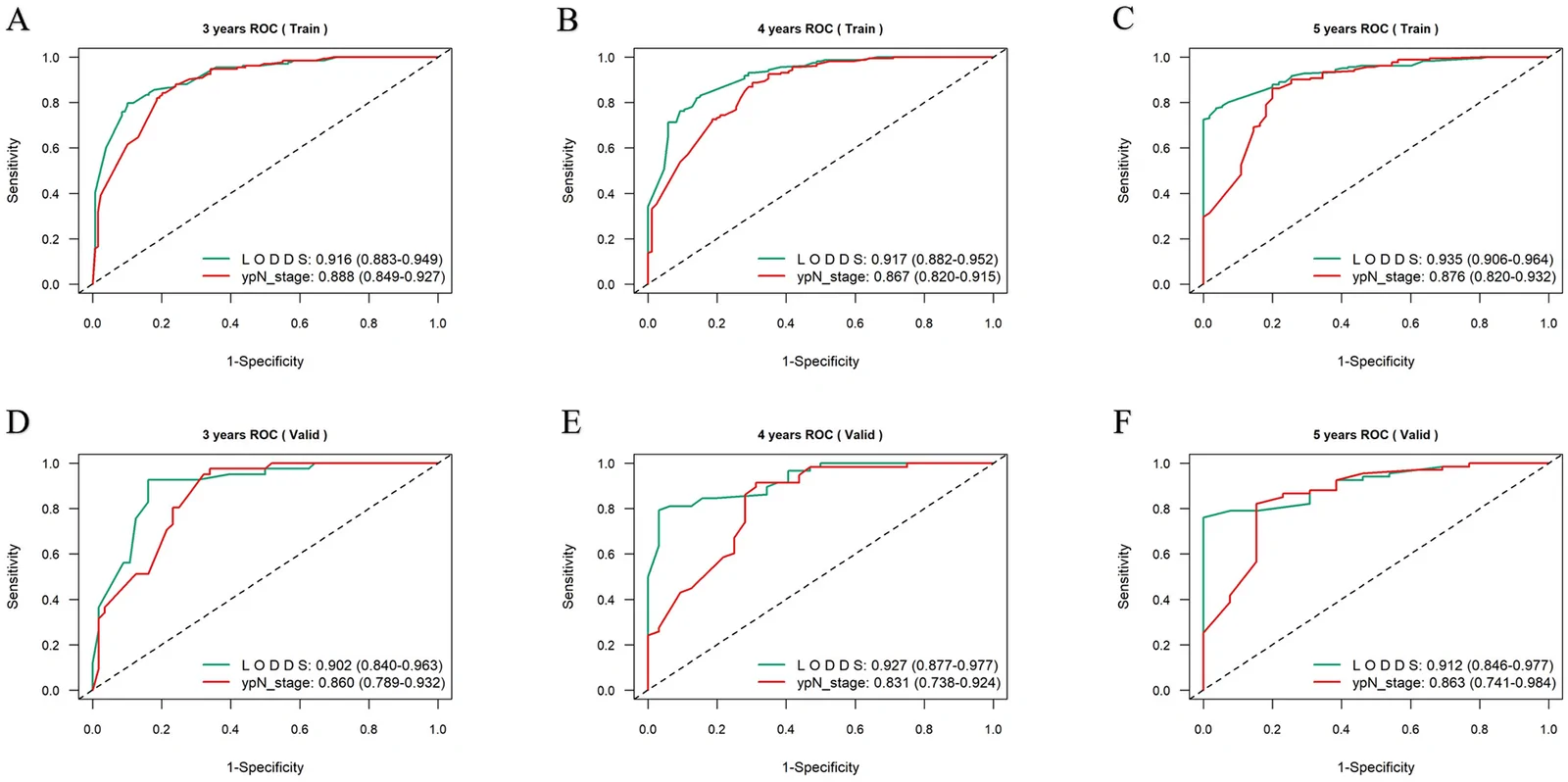
ROC curves of Model 1 and Model 2 for predicting 3-, 4-, and 5-year recurrence in the training and validation cohorts. ypN_stage, post-therapeutic pathological node stage; ROC, Receiver Operating Characteristic.

**Figure 5:**
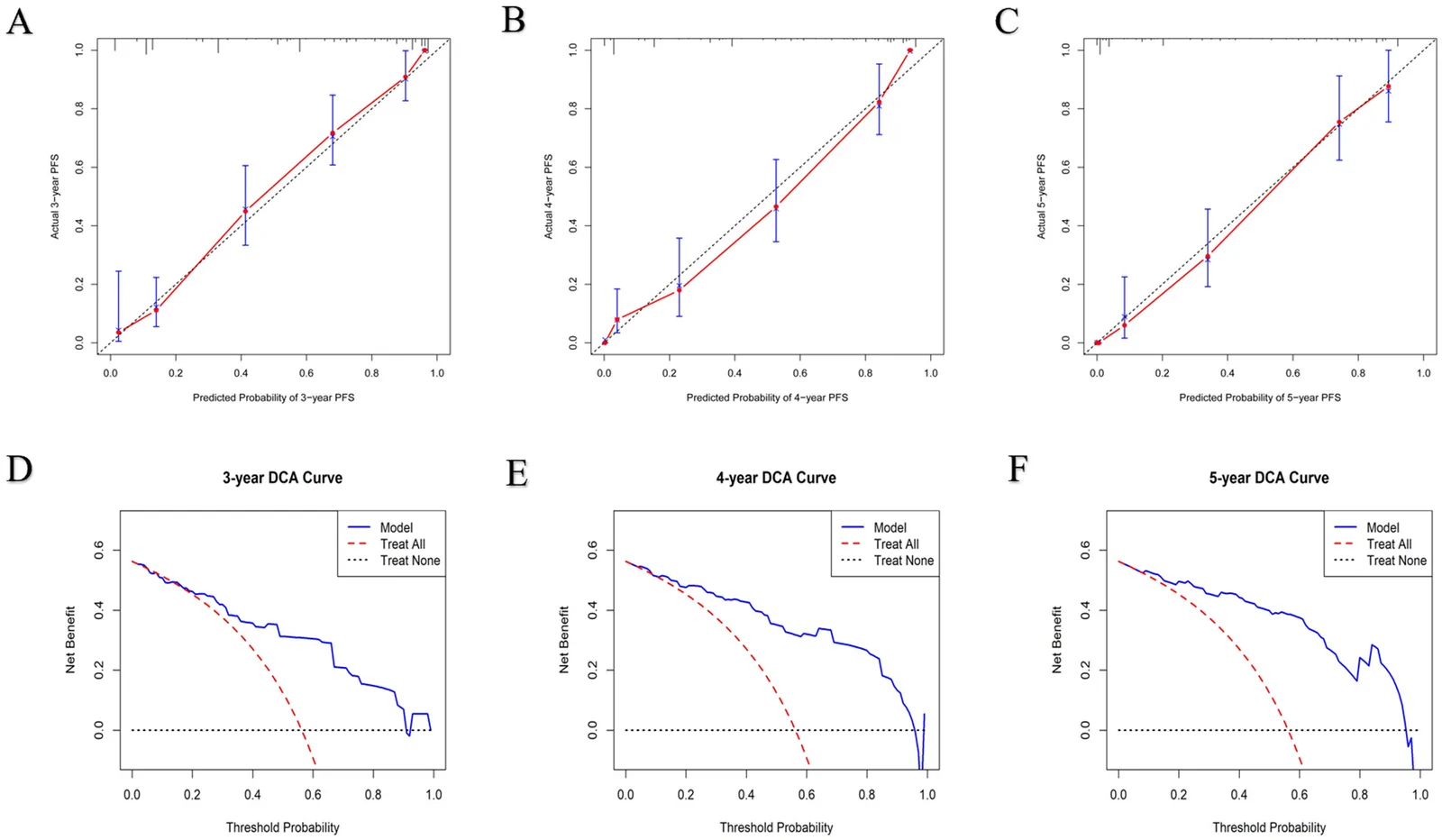
**(A–C)** Calibration curves of Model 1 for predicting 3-, 4-, and 5-year recurrence in the training cohort. **(D–F)** Decision curve analysis of Model 1 for predicting 3-, 4-, and 5-year recurrence in the validation cohort.

Using the final selected variables, two recurrence risk curves for PFS were plotted in the training set (Figures 3A, B), followed by ROC analysis. The discriminative ability of the model was further assessed by comparing the AUC values at 3, 4, and 5 years (Table 3). For model 1 in the training set, the AUC values at 3, 4, and 5 years were 0.916 (95% CI, 0.883–0.949), 0.917 (95% CI, 0.882–0.952), and 0.935 (95% CI, 0.906–0.964), respectively (C–index: 0.843, 95% CI, 0.820–0.866). For model 2, the AUC values at 3, 4, and 5 years were 0.888 (95% CI, 0.849–0.927, DeLong test *p* = 0.045), 0.867 (95% CI, 0.820–0.915, DeLong test *p* = 0.004), and 0.876 (95% CI, 0.820–0.932, DeLong test *p* = 0.005), respectively (C–index: 0.819, 95% CI, 0.792–0.846) (Figures 4A–C). In the validation set, the AUC values for model 1 were 0.902 (95% CI, 0.840–0.963), 0.927 (95% CI, 0.877–0.977), and 0.912 (95% CI, 0.846–0.977), while for model 2, the AUC values were 0.860 (95% CI, 0.789–0.932), 0.831 (95% CI, 0.738–0.924), and 0.863 (95% CI, 0.741–0.984). These findings demonstrate that the model exhibits high predictive efficacy and stability (Figures 4D–F). Calibration curves showed excellent agreement between the predicted and observed survival probabilities at 3, 4, and 5 years (Figures 5A–C). DCA further confirmed that the model provided substantial clinical net benefit within threshold probability ranges of 0.2–0.9 at 3 years, and 0.1–0.9 at 4 and 5 years (Figures 5D–F). Overall, the results indicate that the prognostic model demonstrates robust discriminative ability and predictive performance for PFS.

**Table 3:** Multivariable Cox regression analysis of PFS in the training cohort.

Variable	Model 1		Model 2	
	HR (95% CI)	P-value	HR (95% CI)	*P*-value
Age				
< 60	–	–	–	–
≥60	2.095 (1.358–3.231)	< 0.001^*^	1.528 (1.010–2.312)	0.045^*^
ypT stage				
T1–T2	–	–	–	–
T3–T4	2.088 (1.137–3.833)	0.007^*^	2.067 (1.077–3.967)	0.029^*^
TRG				
0	–	–	–	–
1	2.741 (1.030–7.292)	0.043^*^	2.946 (1.102–7.877)	0.031^*^
2	3.375 (1.176–9.686)	0.024^*^	3.581 (1.192–10.752)	0.023^*^
3	7.708 (2.613–22.735)	< 0.001^*^	8.447 (2.740–26.039)	< 0.001^*^
PNI				
>48.9	–	–	–	–
≤ 48.9	1.817 (1.299–2.540)	0.002^*^	1.882 (1.357–2.610)	< 0.001^*^
LODDS				
< -3.65	–	–	–	–
−3.65 to −0.755	4.307 (2.864–6.476)	< 0.001^*^	–	–
>-0.755	5.299 (3.413–8.226)	< 0.001^*^	–	–
ypN stage				
N0	–	–	–	–
N1	–	–	2.355 (1.146–4.842)	0.020^*^
N2	–	–	2.494 (1.201–5.181)	0.014^*^
N3	–	–	2.961 (1.395–6.287)	0.004^*^

^*^P < 0.05 was considered statistically significant.

### 3.4 Evaluation of the prognostic risk stratification model

**Figure 6:**
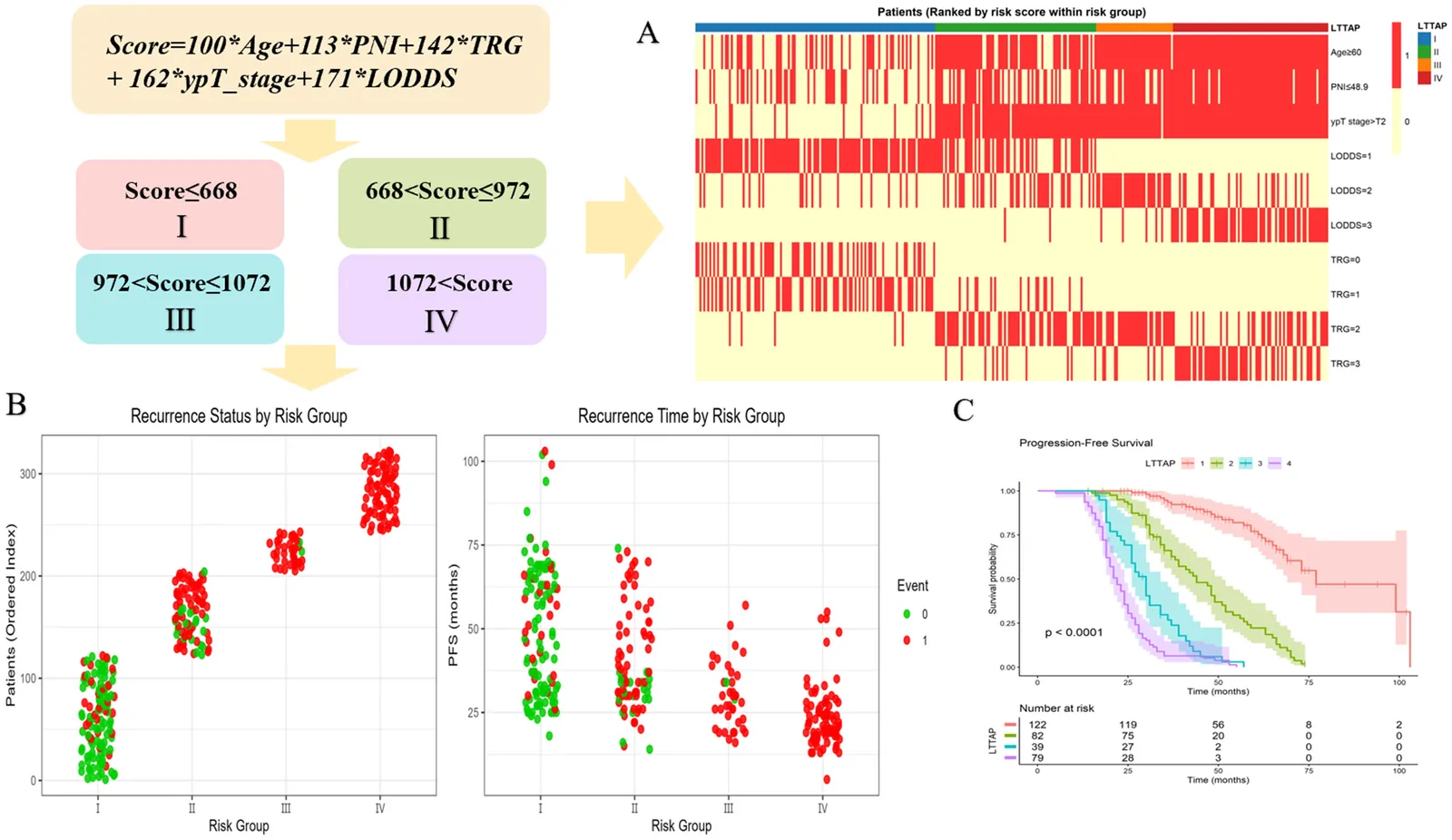
Construction and evaluation of LTTAP. **(A)** Correlation analysis between each predictive factor and the four risk groups. **(B)** Heatmap of survival status and survival time across the four risk groups. **(C)** Survival analysis of the four risk groups.

**Figure 7:**
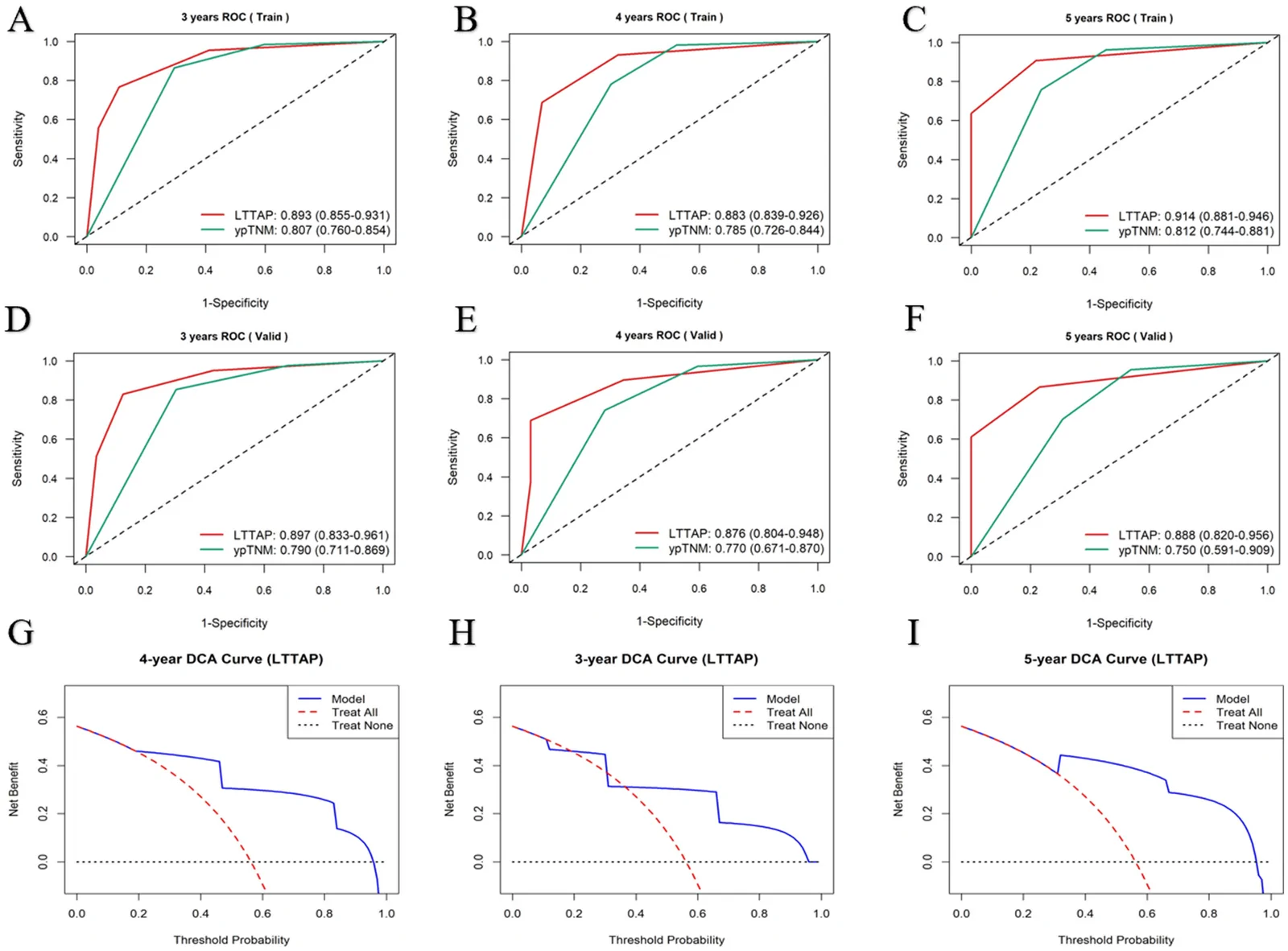
**(A–C)** ROC curves of the LTTAP model and ypTNM stage for predicting 3-, 4-, and 5-year outcomes in the training cohort. **(D–F)** ROC curves of the LTTAP model and ypTNM stage for predicting 3-, 4-, and 5-year outcomes in the validation cohort. **(G–I)** Decision curve analysis of the LTTAP model for predicting 3-, 4-, and 5-year outcomes in the validation cohort.

The feature selection process identified Age, TRG, PNI, LODDS, ypT stage, and ypN stage as independent prognostic factors for NAC-LAGC patients, with the LODDS-based model outperforming the ypN stage model. Utilizing these inflammation-nutritional indices and clinical pathological features, with LODDS as the primary factor, a prognostic risk stratification model (LTTAP) was developed. This model incorporates a risk stratification system based on a scoring formula derived from the PFS nomogram's weight coefficients. The specific formula is as follows: Score = 100 × Age + 113 × PNI + 162 × ypT stage + 142 × TRG + 171 × LODDS. Optimal cut-off points (668, 972, and 1,072) were determined using the “X-tile” package in R software, and patients were classified into four sets: Set I ( ≤ 668), Set II (668 < score ≤ 972), Set III (972 < score ≤ 1,072), and Set IV (1,072 < ). Kaplan–Meier survival analysis revealed significant differences in survival curves among the four patient sets in the training set (*p* < 0.001) (Figure 6C). Further analysis of the relationship between model variables and the risk stratification sets is presented in Figure 6A. Additionally, a comparison of survival status and survival time across different risk sets showed that lower-risk sets generally had better overall conditions, with longer PFS and lower recurrence rates, while higher-risk sets exhibited significantly shorter PFS and higher recurrence probabilities (Figure 6B). To evaluate the discriminative power of the LTTAP compared to traditional TNM staging, ROC analysis was performed in both the training and validation sets. In the training set, the AUC values for the LTTAP at 3, 4, and 5 years were 0.893 (95% CI, 0.855–0.931), 0.883 (95% CI, 0.839–0.926), and 0.914 (95% CI, 0.880–0.946), respectively (C-index: 0.826, 95% CI, 0.803–0.849). For the ypTNM staging in the training set, the AUC values were 0.807 (95% CI, 0.760–0.854), 0.785 (95% CI, 0.726–0.844), and 0.812 (95% CI, 0.744–0.881) (C-index: 0.743, 95% CI, 0.715–0.772) (Figures 7A–C), with Delong test *p*-values all less than 0.05. In the validation set, the AUC values for the LTTAP were 0.897 (95% CI, 0.833–0.961), 0.876 (95% CI, 0.804–0.948), and 0.888 (95% CI, 0.820–0.956), while for the ypTNM staging, the AUC values were 0.790 (95% CI, 0.711–0.869), 0.770 (95% CI, 0.671–0.870), and 0.750 (95% CI, 0.591–0.909) (Figures 7D–F). Clinical decision curves in the validation set indicated that the LTTAP provided significant net clinical benefits across a broad range of threshold probabilities at 3, 4, and 5 years (Figures 7G–I). These results further confirm the reliability and clinical applicability of the LTTAP in prognostic evaluation and risk stratification.

## 4 Discussion

NAC plays a critical role in the treatment of gastric cancer by reducing tumor burden, downstaging the tumor, and increasing the likelihood of achieving R0 resection (2, 22). Previous studies have reported that the R0 resection rate can reach approximately 82% in LAGC patients undergoing NAC (23). However, even in LAGC patients who receive NAC, the postoperative recurrence rate remains high. A study by Glatz et al. (24) demonstrated that despite FLOT chemotherapy and radical surgery, LAGC patients continue to experience distant recurrence, such as peritoneal metastasis. In the present study, as follow-up time increased, recurrences at 3, 4, and 5 years post-surgery among the 450 patients were 174, 218, and 249, respectively. These figures indicate that the postoperative recurrence risk in NAC-LAGC patients not only persists but also worsens over time. To address this challenge, the present study aims to expand on existing literature regarding postoperative recurrence prediction in NAC-LAGC patients by developing a long-term recurrence risk prediction model that incorporates multiple clinical indicators. The findings of this study indicate that advanced age, low prognostic nutritional index (PNI), higher TRG score, high ypT stage, and high LODDS are all significantly associated with postoperative recurrence risk in NAC-LAGC patients. By integrating these multidimensional predictive factors, with LODDS and PNI as key components, a nomogram model was developed to predict long-term recurrence in these patients. To improve the clinical applicability of the model, a simple and easily interpretable risk stratification system was also constructed. This model demonstrated high predictive efficacy in forecasting recurrence risk at 3, 4, and 5 years (Training set AUC: 3 years: 0.893; 4 years: 0.883; 5 years: 0.914).

Individual patient characteristics, particularly age and nutritional status, directly influence chemotherapy tolerance and treatment efficacy. With aging, immune function declines, and the reduction in physical and physiological reserves makes elderly patients more susceptible to chemotherapy intolerance, increasing the risk of adverse reactions, which in turn affects treatment outcomes and tumor recurrence (25). Additionally, NAC often induces gastrointestinal toxicity, directly impairing gastrointestinal function and affecting nutrient absorption. This leads to malnutrition, decreased immune function, and creates an environment conducive to tumor recurrence and metastasis (26). Zhang et al. found that if gastrointestinal toxicity is not effectively managed, patients with gastric and colorectal cancer often experience immune function decline due to persistent malnutrition, further escalating the risk of recurrence and metastasis (27). Tumor-associated malnutrition is frequently accompanied by physical decline, impaired immune function, and enhanced inflammatory responses, all of which contribute to tumor progression and increase the risk of recurrence. Consequently, recent studies have focused on using inflammation–nutrition indices to develop models that predict prognosis and recurrence. Tokunaga's study highlighted the significant predictive value of PNI for postoperative complications, recurrence, and OS in colorectal cancer patients (*p* < 0.001, 95% CI, 2.38–6.89) (28). Wang et al. constructed a model by integrating inflammation–nutrition indices such as the Controlling Nutritional Status score (CONUT) and platelet/lymphocyte ratio (PLR) to predict survival prognosis in cervical cancer patients undergoing postoperative radiotherapy. The model demonstrated AUC values of 0.71, 0.73, and 0.76 for predicting OS at 3, 4, and 5 years, respectively (29). In comparison, this study identified, through multivariate analysis, that advanced age (Age ≥ 60) [HR (95% CI) 2.095 (1.358–3.231), *p* < 0.001] and lower PNI (PNI ≤ 48.9) [HR (95% CI) 1.817 (1.299–2.540), *p* < 0.001] are significant predictors of higher recurrence risk. Therefore, for cancer patients undergoing neoadjuvant therapy, considering nutritional status is critical for improving treatment outcomes and reducing the risk of recurrence.

TRG scoring is a valuable tool for assessing tumor cell regression and is strongly correlated with patient prognosis (30). A higher TRG score indicates chemotherapy resistance or adverse tumor biological behavior associated with invasion and metastasis, with residual tumor burden being more prone to local or distant recurrence (31). ypT staging reflects the residual tumor size and its degree of invasion into surrounding tissues, further indicating the likelihood of recurrence. After neoadjuvant therapy, ypT staging effectively assesses the tumor's biological behavior and prognostic risk (32). For cancer patients undergoing neoadjuvant therapy, the impact of preoperative chemotherapy or radiotherapy on lymph node dissection and staging evaluation during surgery can be significant (33). ypN staging is a critical marker for assessing lymph node metastasis after neoadjuvant therapy. However, incomplete lymph node dissection or difficulties in pathological examination may lead to missed metastasis, impacting the accuracy of staging. In cases where edema persists, lymph nodes may be misclassified as false negatives or positives, further affecting the reliability of clinical prognosis assessments (34).

LODDS, as a comprehensive approach to assessing lymph node burden, has increasingly been incorporated into the prognostic evaluation of gastrointestinal tumors, including gastric cancer, in recent years (16). Lymph nodes are a common route for tumor cell metastasis, with tumor cells spreading to local or distant lymph nodes through the lymphatic system, resulting in lymph node metastasis. Unlike traditional ypN staging, LODDS incorporates both positive and negative lymph nodes, offering a more holistic assessment of lymph node status. This is particularly valuable after neoadjuvant therapy, as LODDS can mitigate the issue of “stage migration” caused by variations in lymph node dissection scope, providing more accurate prognostic predictions (16). For instance, Wang et al. analyzed a SEER cohort of non-small cell lung cancer (NSCLC) patients who underwent surgery after NAC and demonstrated that LODDS exhibited superior survival stratification ability compared to traditional N staging. The AUCs for LODDS in predicting 1-, 3-, and 5-year OS were 0.538, 0.575, and 0.590, respectively, all higher than those for traditional N staging, which were 0.493, 0.538, and 0.549, respectively (35). Although neoadjuvant therapy can inhibit tumor cells and lymph node metastases, lymph node response to treatment is influenced by multiple factors. Lymph node response to neoadjuvant therapy correlates with treatment efficacy, and in some cases, lymph nodes may fail to fully clear tumor cells, resulting in residual metastases that affect recurrence risk. Changes in LODDS not only reflect the impact of neoadjuvant therapy on lymph node metastasis but also provide prognostic insights into the lymph node response, offering a more precise tool for assessing recurrence risk after neoadjuvant therapy in gastric cancer (12, 36). While previous studies have made progress in evaluating short-term prognosis after neoadjuvant therapy for gastric cancer, evidence remains limited regarding the long-term effects of treatment-induced changes in lymph nodes on recurrence risk (37, 38). To address this gap, this study developed a novel and more accurate model for predicting long-term recurrence risk by integrating LODDS, PNI, and other relevant indicators. Our results indicated that model 1 (without ypN staging) exhibited higher AUC values for predictions at 3, 4, and 5 years post-surgery compared to model 2 (without LODDS) (Training set AUC: 3 years: 0.916 vs. 0.888, 4 years: 0.917 vs. 0.867, 5 years: 0.935 vs. 0.876, with Delong test *p*-values < 0.05), suggesting that LODDS provides more precise information for predicting long-term recurrence risk than ypN staging.

Existing prognostic studies primarily focus on pathological indicators. For example, Sun et al. developed a prognostic model integrating ypN staging and TRG grading in LAGC patients undergoing NAC and immunotherapy. This model demonstrated superior predictive ability compared to ypTNM staging (C-index, 0.784 vs. 0.752; *p* < 0.01) (39). Similarly, Li et al. utilized LODDS in their prognostic study of distal cholangiocarcinoma patients, comparing it with other lymph node parameters, and constructed a nomogram model based on LODDS to predict cancer-specific survival (CSS). The model performed well in terms of AUC at 1, 3, and 5 years (AUC = 0.671, 0.705, and 0.665), outperforming the AJCC staging system (C-index: 0.639 vs. 0.577) (40). However, while pathological indicators provide insights into tumor characteristics, they often fail to comprehensively account for systemic factors such as immune status and nutritional condition. When used in isolation, they have limitations and may not fully meet clinical needs for personalized diagnosis and treatment. Existing models, in particular, often overlook non-pathological factors like immune and nutritional status, and variations in lymph node dissection scope can further impact the accuracy of prognostic evaluation. To address these challenges, an increasing number of studies are integrating multiple factors—such as inflammation–nutritional levels, surgical conditions, and tumor characteristics—into composite predictive models (41, 42). In this context, this study introduces LODDS in combination with nutritional and other pathology-related predictive indicators to construct two multidimensional recurrence risk prediction models. By comparing the predictive efficacy of models containing LODDS and ypN staging for evaluating recurrence risk in NAC-LAGC patients, the superiority of LODDS in this area was further validated. The model incorporating LODDS provides a more comprehensive and accurate assessment of long-term recurrence risk. Notably, this model demonstrated excellent performance in predicting 3-year, 4-year, and 5-year recurrence risks (Training set: 3 years AUC = 0.916, 4 years AUC = 0.917, 5 years AUC = 0.935; Validation set: 3 years AUC = 0.902, 4 years AUC = 0.927, 5 years AUC = 0.912). These results indicate that the multidimensional prognostic model developed in this study exhibits high clinical applicability and predictive accuracy.

The LTTAP proposed in this study incorporates multidimensional predictive factors, including clinical pathological characteristics, age, and inflammation-nutrition indices, facilitating a comprehensive assessment of the patient's overall health and treatment response. This approach overcomes the limitations of traditional prognostic evaluation methods, which often fail to account for individual variations and tumor microenvironment influences. The model effectively predicts recurrence risk and enhances clinical decision-making by providing more accurate treatment support. A scoring formula, derived from the weight coefficients of the various indicators in the nomogram, was used to score patients, who were subsequently categorized into different recurrence risk stages based on three cutoff values (668, 972, and 1,072), enabling precise risk stratification for postoperative recurrence in NAC-LAGC patients. The results demonstrated that the LTTAP outperformed ypTNM staging in predicting recurrence risk at 3, 4, and 5 years (Training set: 3 years: 0.893 vs. 0.807, 4 years: 0.883 vs. 0.785, 5 years: 0.914 vs. 0.812; Validation set: 3 years: 0.897 vs. 0.790, 4 years: 0.876 vs. 0.770, 5 years: 0.888 vs. 0.750). In conclusion, LTTAP provides valuable guidance for clinical practice, offering more accurate predictions of recurrence risk in NAC-LAGC patients than ypTNM staging. It holds substantial clinical significance for guiding personalized postoperative follow-up strategies and optimizing decision-making in gastric cancer.

However, despite the favorable predictive performance of the models, several limitations should be acknowledged. First, the retrospective, single-center design may have introduced selection bias and limited the generalizability of the findings; therefore, prospective multicenter validation is warranted. Second, owing to the long study period from 2016 to 2025, information on Lauren classification, treatment-related adverse events, and postoperative adjuvant treatment was incompletely and inconsistently documented. Moreover, NAC-induced histomorphological changes may have reduced the reliability of postoperative Lauren classification. Consequently, the prognostic effects of these factors could not be fully evaluated, potentially resulting in residual confounding and limiting the comprehensiveness of the models.

## 5 Conclusions

In conclusion, we have developed and validated a powerful prognostic risk stratification model (LTTAP) that integrates multidimensional predictive factors, including LODDS, PNI, pathological characteristics, and age, to estimate the long–term recurrence risk in NAC–LAGC patients. This model provides reliable risk stratification results and is expected to become a practical tool to support individualized postoperative follow–up strategies and management in clinical practice.

## Data Availability

The raw data supporting the conclusions of this article will be made available by the authors, without undue reservation.
